# Delayed Clinical Manifestation of Malignant Potential in a Cortisol‐Secreting Adrenal Tumor Initially Diagnosed as Adenoma

**DOI:** 10.1002/iju5.70166

**Published:** 2026-03-16

**Authors:** Fumihiro Ito, Koki Kobayashi, Gaku Hayashi, Shunsuke Kamijo, Takashi Fujita

**Affiliations:** ^1^ Department of Urology Gifu Prefectural Tajimi Hospital Tajimi Japan

**Keywords:** adrenocortical carcinoma, capsular invasion, cortisol‐secreting adrenal tumor, long‐term follow‐up, malignant potential

## Abstract

**Introduction:**

Distinguishing adrenocortical carcinoma (ACC) from adenoma is often difficult, particularly in hormonally active tumors with borderline histologic features.

**Case Presentation:**

A 55‐year‐old woman presented with abdominal distension and central obesity. Hormonal evaluation confirmed ACTH‐independent Cushing's syndrome. Imaging revealed a 60‐mm left adrenal mass. Laparoscopic adrenalectomy was performed; pathology was initially interpreted as cortical adenoma with mild nuclear atypia and a Ki‐67 labeling index of 6%. The patient remained disease‐free until year three postoperatively when rising cortisol levels and surveillance imaging revealed a 36‐mm retroperitoneal mass. Open nephrectomy and tumor excision were performed. Histopathology confirmed ACC. Retrospective review of the initial specimen revealed features consistent with early carcinoma.

**Conclusion:**

This case illustrates that cortisol‐producing adrenal tumors initially diagnosed as adenomas may harbor unrecognized malignant potential, which can become clinically evident several years after surgery.

## Keynote Message

Functional adrenal tumors initially diagnosed as adenomas may harbor latent malignant potential despite benign histologic features. This case highlights the limitations of morphology‐based classification alone and underscores the importance of careful pathological review and prolonged postoperative surveillance.

**Abbreviations**
ACC: adrenocortical carcinoma
CT: computed tomography
FDG: fluorodeoxyglucose
Ki‐67: Ki‐67 labeling index
MRI: magnetic resonance imaging
PET: positron emission tomography

## 1 Introduction

Adrenocortical tumors range from benign adenomas to aggressive carcinomas, and distinguishing them remains challenging despite the Weiss system, particularly in functional or borderline tumors [1, 2, 3, 4, 5]. Functional tumors carry a higher malignant risk than nonfunctioning adenomas, and low Ki‐67 does not exclude malignancy [6, 7, 8, 9, 10, 11, 12].

We present a rare case of a cortisol‐producing adrenal tumor initially diagnosed as a benign adenoma that developed a local recurrence nearly 4 years after adrenalectomy and was subsequently confirmed as adrenocortical carcinoma (ACC). This case highlights the difficulty of distinguishing truly benign adenomas from cortisol‐producing tumors with latent malignant potential at the initial diagnosis and underscores the need for prolonged follow‐up.

## 2 Case Presentation

A 55‐year‐old woman was referred for investigation of abdominal fullness and central obesity. She had a medical history of diabetes and dyslipidemia. Hormonal evaluation demonstrated ACTH‐independent Cushing's syndrome, with elevated serum and urinary cortisol levels, loss of diurnal variation, and failure of dexamethasone suppression.

**FIGURE 1:**
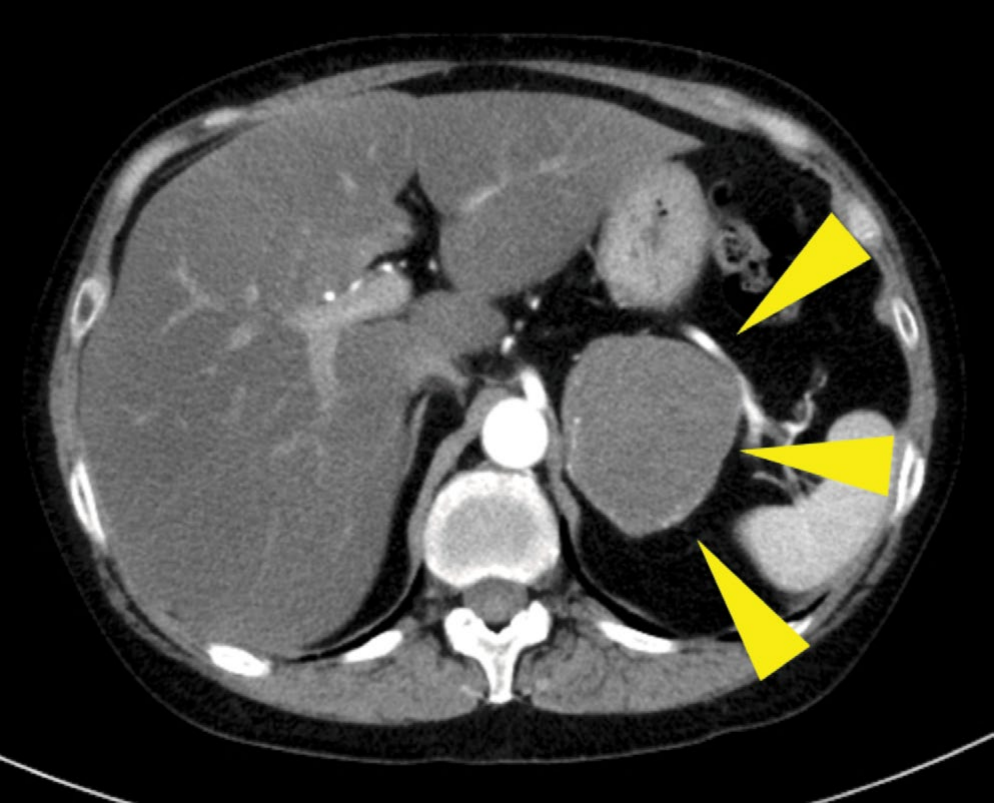
Initial contrast‐enhanced CT demonstrating a well‐demarcated 60‐mm left adrenal mass without surrounding organ invasion.

Adrenal scintigraphy showed uptake in the left adrenal gland with suppression of the contralateral side. MRI showed a well‐demarcated 60‐mm mass in the left adrenal gland without evidence of local invasion (Figure 1). The patient underwent laparoscopic transperitoneal adrenalectomy in February 2022. Operative time was 1 h 59 min with minimal blood loss, and there were no perioperative complications.

## 3 Initial Pathology

**FIGURE 2:**
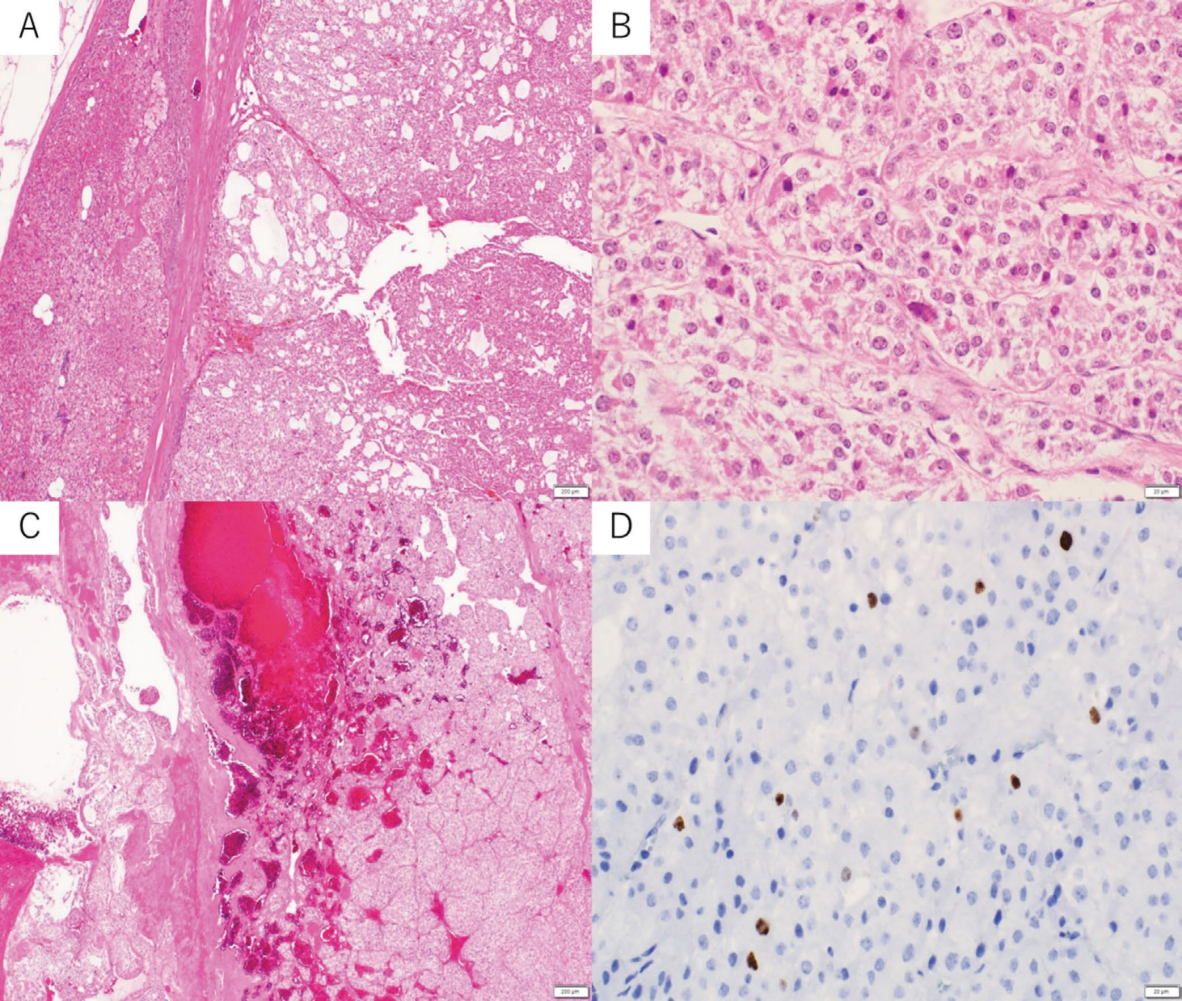
Histopathologic findings of the initial adrenal tumor. (A) Low‐power view showing a relatively well‐demarcated tumor adjacent to normal adrenal cortex. (B) High‐power view demonstrating tumor cells with mild nuclear atypia and eosinophilic cytoplasm. (C) Focal intratumoral hemorrhage without evidence of capsular or vascular invasion. (D) Ki‐67 immunohistochemical staining (hotspot) showing a labeling index of approximately 6%. (Hematoxylin and eosin staining in A–C.)

The resected specimen measured 60 × 55 mm. Histopathologic examination demonstrated a relatively well‐demarcated tumor adjacent to normal adrenal cortex (Figure 2A). Tumor cells showed mild to moderate nuclear atypia without atypical mitoses (Figure 2B). No tumor necrosis, capsular invasion, or vascular invasion was identified (Figure 2C). The mitotic count was 0 per 50 high‐power fields. The Ki‐67 labeling index (hotspot) was 6% (Figure 2D).

Retrospective reassessment using the Weiss criteria demonstrated that the primary tumor fulfilled three of nine parameters: nuclear atypia, < 25% clear cells, and diffuse architecture (Weiss score 3/9). Mitotic rate was 0/50 HPF, with no necrosis or invasion. The Helsinki score was 6.

At initial evaluation, the absence of mitotic activity, necrosis, and invasive features supported a diagnosis of adenoma. Despite meeting the Weiss threshold retrospectively, the absence of necrosis, mitoses, and invasion led to a benign interpretation.

## 4 Postoperative Course

The patient was started on hydrocortisone replacement and tapered over 2 years. Hormonal surveillance showed sustained cortisol suppression for over 2 years.

**FIGURE 3:**
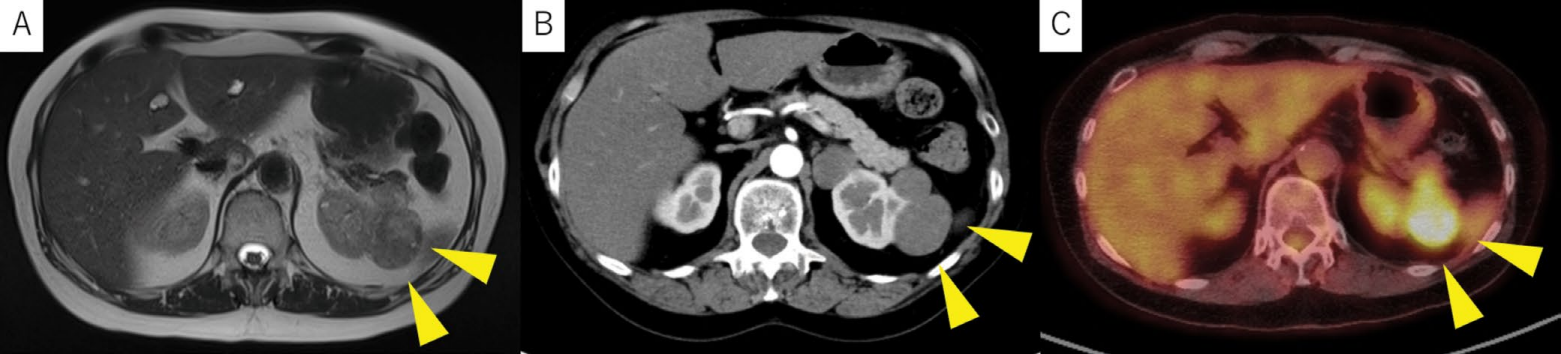
Imaging of recurrent tumor. (A) Magnetic resonance imaging showing a 36‐mm solid mass in the left renal bed with heterogeneous signal intensity on T2‐weighted imaging. (B) Contrast‐enhanced CT demonstrating heterogeneous enhancement of the lesion. (C) PET‐CT showing moderate FDG uptake in the recurrent mass.

Surveillance MRI every 6 months revealed no recurrence until July 2024. In September 2025, an abdominal MRI for unrelated pancreatic follow‐up identified a 36‐mm mass in the left renal bed (Figure 3A). The patient was asymptomatic, and the MRI showed a heterogeneous T2 signal.

Subsequent contrast‐enhanced CT showed a solid mass with progressive enhancement, suggestive of recurrence (Figure 3B). PET‐CT showed moderate FDG uptake in the same region (Figure 3C). Hormonal testing confirmed cortisol elevation (12.8 μg/dL post‐dexamethasone suppression test) and persistent ACTH suppression.

## 5 Second Surgery and Pathology

In November 2025, the patient underwent an open transperitoneal left nephrectomy with tumor resection. The mass was tightly adherent to the kidney but separate from the pancreas and spleen. No lymphadenectomy was performed.

**FIGURE 4:**
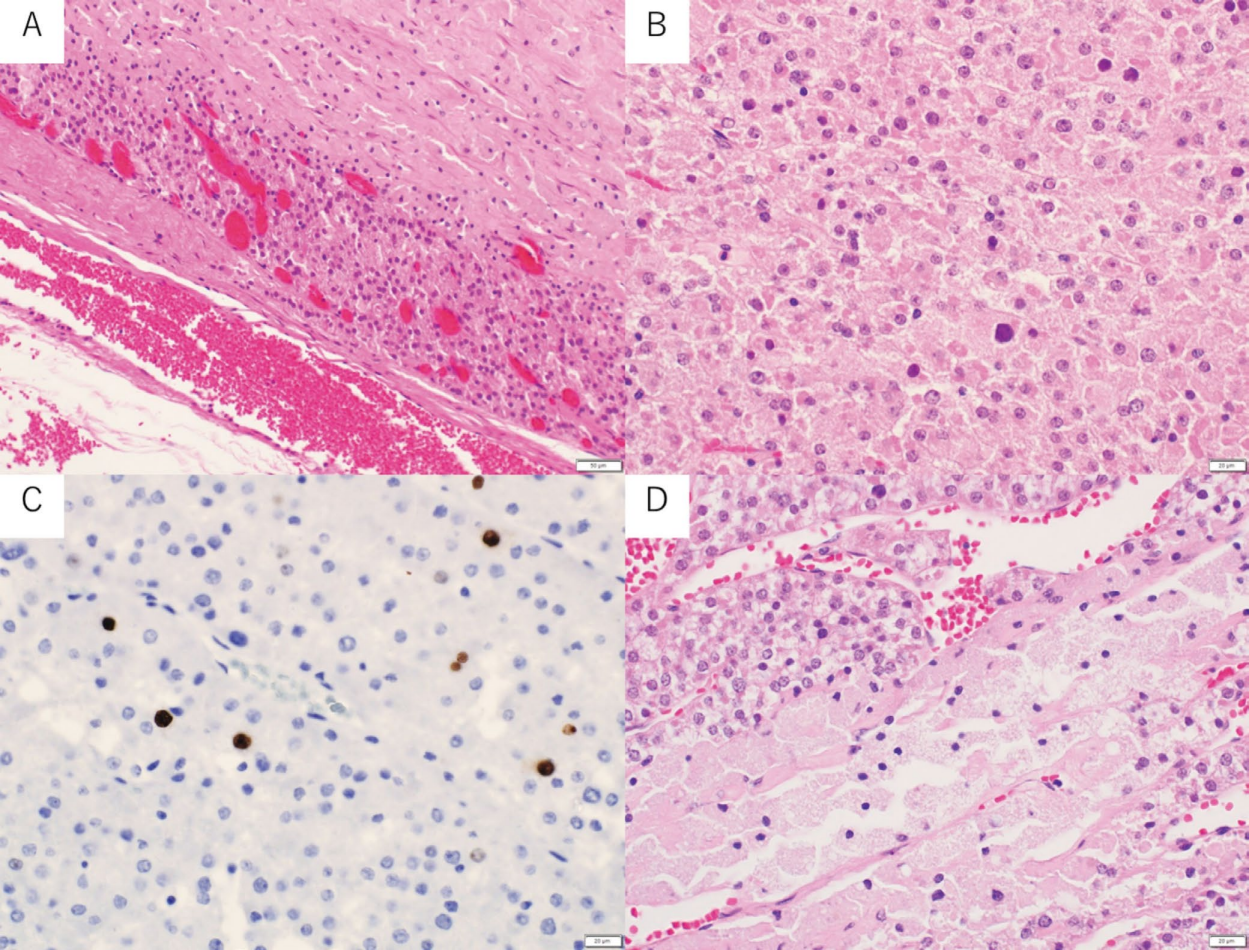
Pathology of the recurrent mass confirming adrenocortical carcinoma. (A) Low‐power view showing tumor cells extending to and focally breaching the tumor capsule, suspicious for capsular invasion. (B) High‐power view demonstrating tumor cells with increased nuclear atypia compared with the primary lesion. (C) Immunohistochemical staining for Ki‐67 (MIB‐1) showing a low labeling index of approximately 4%. (D) Tumor necrosis within the recurrent lesion. (Hematoxylin and eosin staining in A, B, and D.)

Histopathologic examination demonstrated capsular invasion (Figure 4A) and tumor necrosis (Figure 4D). Nuclear atypia was present (Figure 4B), whereas mitotic figures were not identified (0/50 high‐power fields), and vascular invasion was absent. The Ki‐67 labeling index (hotspot) was 4% (Figure 4C).

The recurrent tumor fulfilled four Weiss criteria—nuclear atypia, < 25% clear cells, tumor necrosis, and capsular invasion—corresponding to a Weiss score of 4/9. The mitotic rate was 0 per 50 high‐power fields, and vascular invasion was not identified. The Helsinki score was 7. These findings supported the diagnosis of ACC.

## 6 Discussion

ACC is a rare but aggressive malignancy with heterogeneous clinical behavior [13]. This case illustrates important diagnostic considerations regarding the malignant potential of functional adrenal tumors. Although the primary tumor lacked mitotic activity, necrosis, and invasive features, retrospective scoring revealed a Weiss score of 3/9, placing the lesion at the diagnostic threshold for malignancy. However, the subsequent development of recurrent ACC raises the key question of whether malignant potential was acquired over time or underestimated at the initial diagnosis, a distinction that has direct implications for postoperative surveillance.

### 6.1 Functional Status as a Malignant Risk Factor

Cortisol‐secreting tumors are more frequently associated with malignancy than nonfunctioning adenomas [6, 7]. In our case, ACTH‐independent Cushing's syndrome was present despite histology below carcinoma thresholds, and recurrence underscores that functionality warrants suspicion even when morphology appears benign.

### 6.2 Histologic Criteria May Underestimate Malignant Potential

Although the Weiss system remains the standard diagnostic framework for adrenocortical tumors, previous reports have highlighted diagnostic uncertainty in borderline or atypical lesions [14, 15, 16, 17].

Retrospective reassessment showed that the primary tumor fulfilled three Weiss criteria (3/9). Despite low mitotic activity and Ki‐67 (6%), it met the diagnostic threshold for malignancy, illustrating the limitations of morphology‐based classification in borderline tumors.

The recurrent lesion demonstrated an increased Weiss score (4/9) due to the emergence of necrosis and capsular invasion. Notably, mitotic activity remained absent and the Ki‐67 index remained low, indicating that malignant behavior may occur despite low proliferative indices.

Collectively, these findings suggest that the primary lesion most likely represented an early‐stage carcinoma underestimated at initial evaluation rather than a benign adenoma.

### 6.3 Importance of Complementary Diagnostic Markers

Integration of proliferative indices, reticulin evaluation, and the Helsinki score may improve prognostic accuracy beyond classical systems [8, 10, 11, 12, 18]. Adjunctive markers and expert pathology review can reduce misclassification in borderline cases.

### 6.4 Long‐Term Follow‐Up Is Essential, Especially in Functional or Borderline Tumors

ENSAT studies underscore the importance of long‐term follow‐up in ACC [19, 20]. Standardized surveillance strategies for large functional tumors initially diagnosed as adenomas remain undefined. This case suggests that prolonged hormonal and radiologic follow‐up may be warranted in such patients [6, 7].

In our case, recurrence occurred nearly 4 years after adrenalectomy despite initially benign pathology, consistent with reports of delayed recurrence in borderline adrenal tumors [8, 9, 10, 11, 12]. These findings support surveillance beyond 5 years in functional or borderline tumors.

### 6.5 Clinical Implications

Functional adrenal tumors with subtle atypia should not be assumed to be benign. Borderline cases warrant multidisciplinary review with adjunctive markers (Ki‐67 hotspots, PHH3, reticulin), and long‐term hormonal and radiologic surveillance is essential even when initial histology appears benign. Recurrence should prompt reevaluation of the original specimen, as malignant features may become clearer retrospectively.

## 7 Conclusion

We report a rare case in which a cortisol‐producing adrenal tumor initially diagnosed as an adenoma later manifested clinically aggressive behavior consistent with ACC. This case underscores the limitations of morphology‐based classification and the need for careful pathology review and prolonged surveillance in functional adrenal tumors.

## Ethics Statement

According to the policy of our institution, formal IRB review is not required for single‐patient case reports that do not disclose identifiable private information.

## Consent

Written informed consent for publication, including images, was obtained from the patient (or her legal representative) and is available to the Editorial Office upon request.

## Conflicts of Interest

The authors declare no conflicts of interest.

## Data Availability

Data sharing not applicable to this article as no datasets were generated or analysed during the current study.

## References

[1] L. M. Weiss , “Comparative Histologic Study of 43 Metastasizing and Nonmetastasizing Adrenocortical Tumors,” American Journal of Surgical Pathology 8, no. 3 (1984): 163–169, 10.1097/00000478-198403000-00001. 6703192

[2] L. M. Weiss , L. J. Medeiros , and A. L. Vickery, Jr. , “Pathologic Features of Prognostic Significance in Adrenocortical Carcinoma,” American Journal of Surgical Pathology 13, no. 3 (1989): 202–206, 10.1097/00000478-198903000-00004. 2919718

[3] M. Fassnacht , W. Arlt , I. Bancos , et al., “Management of Adrenal Incidentalomas: European Society of Endocrinology Clinical Practice Guideline,” European Journal of Endocrinology 175, no. 2 (2016): G1–G34, 10.1530/EJE-16-0467. 27390021

[4] M. Fassnacht , O. M. Dekkers , T. Else , et al., “European Society of Endocrinology Clinical Practice Guidelines on the Management of Adrenocortical Carcinoma in Adults,” European Journal of Endocrinology 179, no. 4 (2018): G1–G46, 10.1530/EJE-18-0608. 30299884

[5] R. Libé , “Adrenocortical Carcinoma (ACC): Diagnosis, Prognosis, and Treatment,” Frontiers in Cell and Developmental Biology 3 (2015): 45, 10.3389/fcell.2015.00045. 26191527 PMC4490795

[6] M. Fassnacht , M. Kroiss , and B. Allolio , “Update in Adrenocortical Carcinoma,” Journal of Clinical Endocrinology and Metabolism 98, no. 12 (2013): 4551–4564, 10.1210/jc.2013-3020. 24081734

[7] M. Terzolo , A. Angeli , M. Fassnacht , et al., “Adjuvant Mitotane Treatment for Adrenocortical Carcinoma,” New England Journal of Medicine 356, no. 23 (2007): 2372–2380, 10.1056/NEJMoa063360. 17554118

[8] Y. C. Huang , Y. C. Tsai , K. B. Tsai , et al., “Oncocytic Adrenocortical Neoplasm With Delayed Metastasis Revealed by Ki‐67 and PHH3 Hotspot Analysis,” Medicina (Kaunas, Lithuania) 58, no. 6 (2022): 900, 10.3390/medicina58060900.35888619

[9] J. Yoo , J. H. Park , H. J. Jeong , et al., “Adrenal Cortical Neoplasm of Uncertain Malignant Potential Arising in Heterotopic Adrenal Tissue,” Journal of Pathology and Translational Medicine 53, no. 5 (2019): 296–299, 10.4132/jptm.2019.07.09. PMC6435991 30472817

[10] E. Duregon , M. Volante , J. Giorcelli , et al., “Prognostic Performances of PHH3 Mitotic Count and Ki‐67 Index in Adrenocortical Carcinoma,” Modern Pathology 27, no. 9 (2014): 1246–1254, 10.1038/modpathol.2013.242. 24434900

[11] F. Beuschlein , J. Weigel , W. Saeger , et al., “Major Prognostic Role of Ki67 in Localized Adrenocortical Carcinoma After Complete Resection,” Journal of Clinical Endocrinology and Metabolism 100, no. 3 (2015): 841–849, 10.1210/jc.2014-3182. 25559399

[12] R. Morimoto , F. Satoh , O. Murakami , et al., “Ki67/MIB1 Labeling Index as a Predictor for Recurrence of Adrenocortical Carcinomas,” Endocrine Journal 55, no. 1 (2008): 49–55, 10.1507/endocrj.K07-110. 18187873

[13] L. A. Erickson , M. Rivera , and J. Zhang , “Adrenocortical carcinoma: review and update,” Advances in Anatomic Pathology 21, no. 3 (2014): 151–159, 10.1097/PAP.0000000000000025. 24713984

[14] M. Bisceglia , O. Ludovico , A. Di Mattia , et al., “Adrenocortical Oncocytic Tumors: Report of 10 Cases and Literature Review,” International Journal of Surgical Pathology 12, no. 3 (2004): 231–243, 10.1177/106689690401200304. 15306935

[15] L. Mearini , R. Del Sordo , E. Costantini , E. Nunzi , and M. Porena , “Adrenal Oncocytic Neoplasm: A Systematic Review,” Urologia Internationalis 91, no. 2 (2013): 125–133, 10.1159/000350440. 23147196

[16] B. T. Y. Lin , S. M. Bonsib , G. W. Mierau , L. M. Weiss , and L. J. Medeiros , “Oncocytic Adrenocortical Neoplasms: Report of Seven Cases and Literature Review,” American Journal of Surgical Pathology 22, no. 5 (1998): 603–614, 10.1097/00000478-199805000-00008. 9591731

[17] C. P. Chin , R. Grauer , B. Ucpinar , et al., “Oncocytic Adrenocortical Neoplasm of Borderline Uncertain Malignant Potential Diagnosed After Robotic Adrenalectomy,” BMC Urology 23 (2023): 60, 10.1186/s12894-023-01238-1. 37061691 PMC10105432

[18] E. Duregon , R. Cappellesso , V. Maffeis , et al., “Reticulin Algorithm for Adrenocortical Tumors: A Literature Review,” Endocrine Pathology 31, no. 2 (2020): 118–126, 10.1007/s12022-020-09622-1.

[19] A. Berruti , M. Fassnacht , E. Baudin , et al., “Adjuvant Therapy for Adrenocortical Carcinoma: A Position Statement,” Journal of Clinical Oncology 28, no. 23 (2010): e401–e402, 10.1200/JCO.2009.27.8426. 20567001

[20] R. Libé , I. Borget , C. L. Ronchi , et al., “Prognostic Factors in Stage III–IV Adrenocortical Carcinomas: An ENSAT Study,” Annals of Oncology 26, no. 10 (2015): 2119–2125, 10.1093/annonc/mdv329. 26392430

